# 4-(4-Hy­droxy­phenyl)-2,2,4-trimethyl-7,8-benzo­thia­chroman, a fused-ring counterpart of *thia*-Dianin’s compound

**DOI:** 10.1107/S2056989017014608

**Published:** 2017-10-20

**Authors:** Christopher S. Frampton, Joseph J. McKendrick, David D. MacNicol

**Affiliations:** a Wolfson Centre for Materials Processing, Brunel University London, Kingston Lane, Uxbridge UB8 3PH, England; b School of Chemistry, University of Glasgow, Glasgow G12 8QQ, Scotland

**Keywords:** crystal structure, *thia*-Dianin’s compound, hydrogen bonding

## Abstract

The title compound, C_22_H_22_OS [systematic name: 4-(1,3,3-trimethyl-2,3-di­hydro-1*H*-4-thia­phenanthren-1-yl)phenol], crystallizes unsolvated from nitro­methane as colourless prisms (m.p. 425–427 K) in the polar monoclinic space group *Ia* with *Z*′ = 2 (mol­ecules *A* and *B*). Both independent mol­ecules possess a very similar *proximal* conformation, this referring to the juxtaposition of the 4-hy­droxy­phenyl substituent with respect to the *syn*-related methyl group. In the crystal, mol­ecule *A* is linked to mol­ecule *B* by an O—H⋯O hydrogen bond. In turn, mol­ecule *B* exhibits a weak O—H⋯π inter­action with the phenolic group of mol­ecule *A* related by *a*-glide symmetry. Together, these lead to [100] chains.

## Abstract 2

In the title compound, both independent mol­ecules possess a very similar *proximal* conformation, this referring to the juxtaposition of the 4-hy­droxy­phenyl substituent with respect to the *syn*-related methyl group.

## Chemical context   

As part of a detailed study of clathrate formation by systems related to Dianin’s compound (Frampton *et al.*, 2013 ▸, 2017*a* ▸,*b* ▸; MacNicol, 1984 ▸), we have investigated structural modifications of *thia*-Dianin’s compound **2**, the direct thia­chroman counterpart of Dianin’s compound itself, **3**. This led to inter­esting and diverse outcomes: (i) oxidation of **2** gave the colourless and beautifully crystalline sulfone **4**, which crystallises in the polar space group *Cc* with *Z*′ = 1; (ii) crystals of **4** exhibit a significant second-harmonic generation (SHG) effect (Frampton *et al.*, 1992 ▸); (iii) introduction of a methyl group at position carbon-7 led to spontaneous resolution with a structure in *P*2_1_2_1_2_1_, *Z′* = 1; and (iv) introduction of a methyl group at either the 6- or 8-position yielded new clathrate systems isomorphous with **2** and **3**, space group *R* 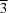 (Hardy *et al.*, 1979 ▸). The latter clathrate networks are comprised of columns formed from infinite stacking of hexa­meric hydrogen-bonded [OH]_6_ units along the *c*-axial direction, with clathrate formation being dependent upon efficient packing with adjacent threefold screw-related columns. Compound **1** was prepared to establish the effect on the resulting crystal packing of substanti­ally extending the mol­ecular skeleton of **2**; the introduction of the bulky benzo group was expected to cause serious disruption to the inter­column packing.

## Structural commentary   

**Figure 1:**
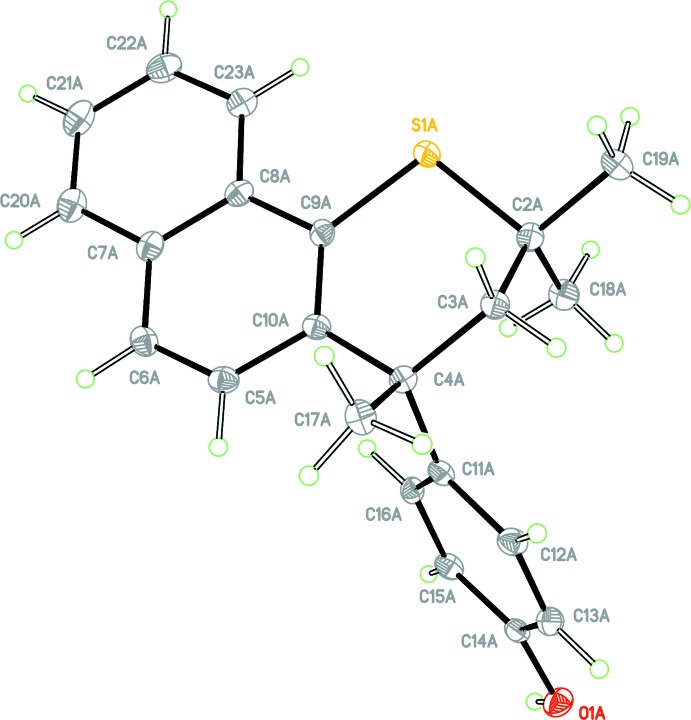
View of mol­ecule *A* of the asymmetric unit with the atom labelling. Displacement ellipsoids are drawn at the 50% probability level.

**Figure 3:**
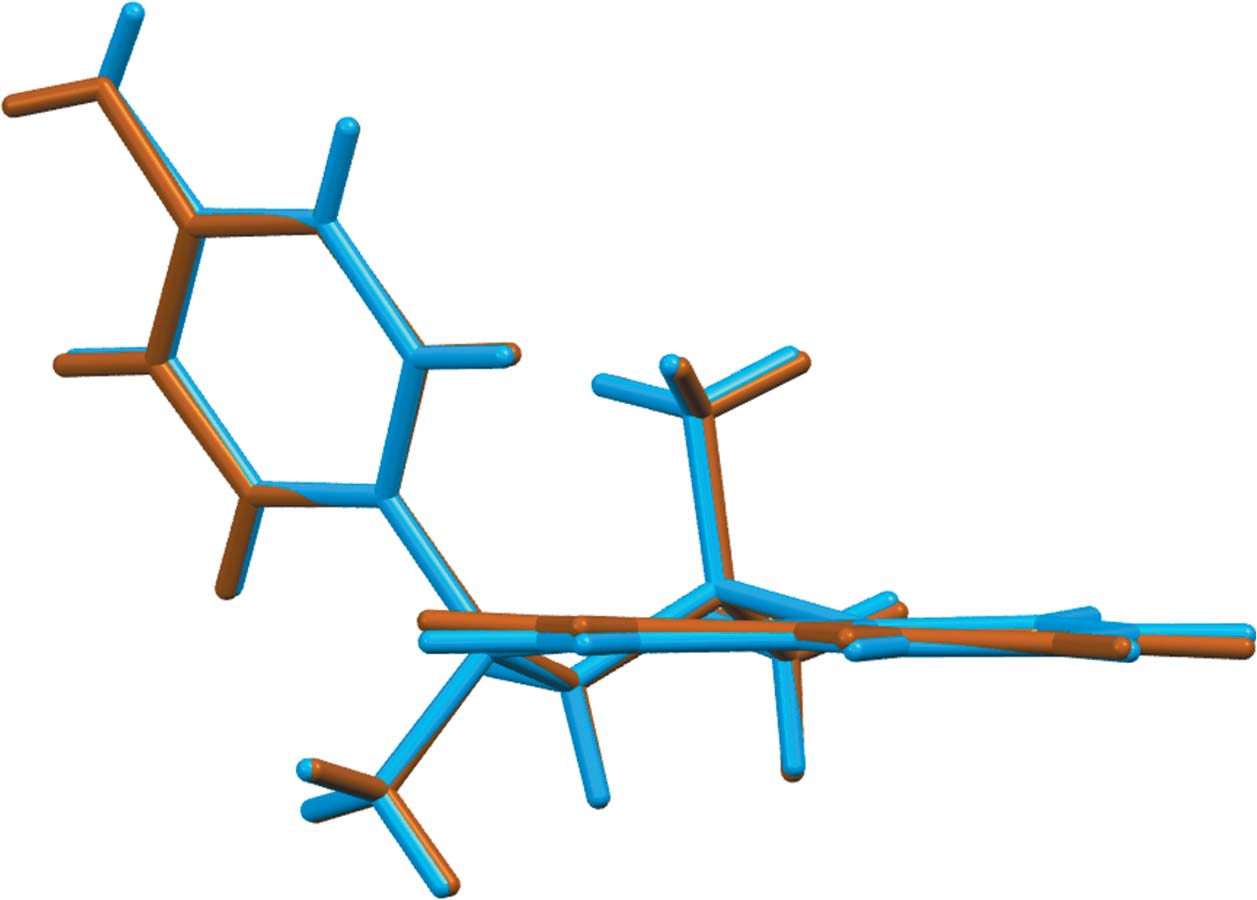
View of the overlay of mol­ecule *A* (blue) and mol­ecule *B* (brown).

The crystal structure of **1** is monoclinic, space group *Ia*, with two independent mol­ecules in the asymmetric unit (*Z*′ = 2). For clarity, each independent mol­ecule is labelled with the suffix *A* or *B*, respectively. Figs. 1 ▸ and 2 ▸ show displacement ellipsoid plots for the two independent mol­ecules. Both independent mol­ecules possess a very similar *proximal* conformation, this referring to the juxtaposition of the 4-hy­droxy­phenyl substituent with respect to the *syn*-related methyl group. The C2—C3—C4—C11 torsion angles for mol­ecules *A* and *B* are 79.5 (4) and 81.4 (4)°, respectively; the corresponding torsion angle for racemic Dianin’s compound has magnitude 80.67° (Lee *et al.*, 2014 ▸). The expected torsion angle value for a *distal* conformation is ∼160°. The torsion angle S1—C2—C3—C4, defining the heterocyclic ring chirality, has values of 62.8 (3) and 63.3 (3)° for *A* and *B*, respectively. Fig. 3 ▸ shows an overlay (Macrae *et al.*, 2008 ▸) of mol­ecules *A* and *B* shown in blue and brown, respectively, with an r.m.s. displace­ment of 0.0789 Å. In addition to showing the *proximal* conformation of both mol­ecules, it can be seen that the two mol­ecules differ only in the directional orientation of the phenolic H atom. The dihedral angles between the naphthalene C5–C10/C20–C23 ring system and the C11–C16 phenol ring are 74.25 (9) and 70.57 (9)° for mol­ecules *A* and *B*, respectively. It is clear that the addition of the fused benzo ring to the *thia*-Dianin framework across positions C7 and C8 has caused significant disruption to the inter­column packing to prevent formation of the conventional *R* 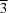 host lattice.
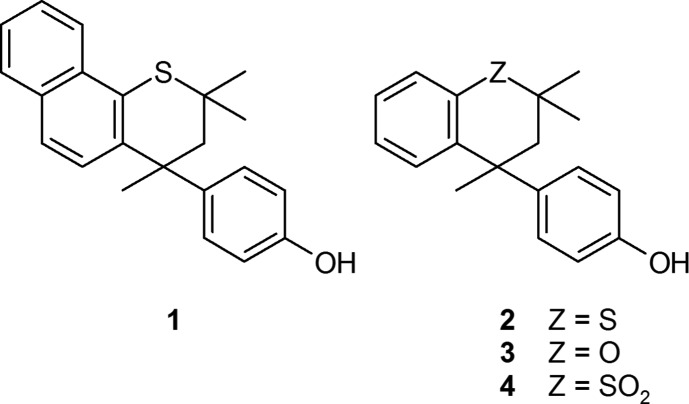


## Supra­molecular features   

**Figure 4:**
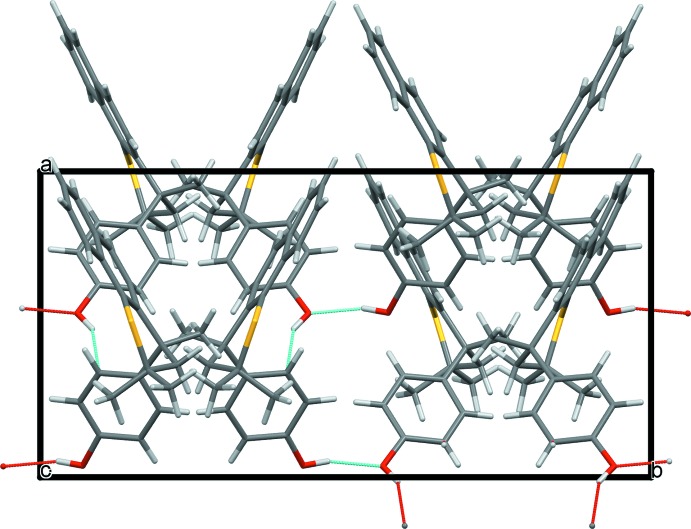
View of the crystal packing down the *c* axis. O—H⋯O and O—H⋯π hydrogen bonds are shown as red and blue dotted lines (see Table 1 ▸ and text).

**Figure 5:**
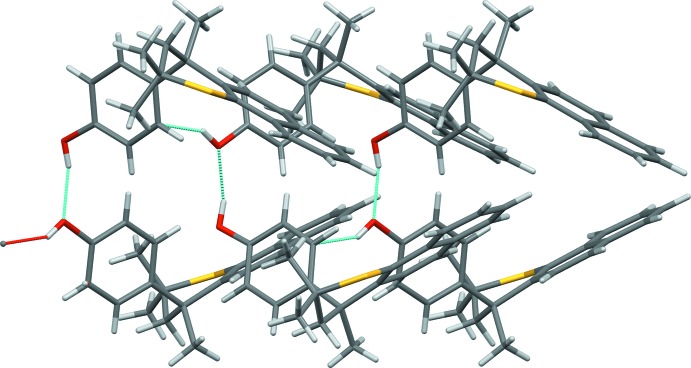
View of the hydrogen-bonded chain that propagates along the *a* axis of the crystal. The O—H⋯O and O—H⋯π hydrogen bonds are shown as red and blue dotted lines and the view is down the *c* axis.

A view of the crystal packing down the *c* axis is shown in Fig. 4 ▸. In the crystal the two independent mol­ecules in the asymmetric unit, *A* and *B*, are linked by an O—H⋯O hydrogen bond (Table 1 ▸). Mol­ecule *B* exhibits a weak O—H⋯π inter­action, shortest length H1*B*⋯C16*A* = 2.54 (6) Å (this being some 0.35 Å less than the Pauling sum of the van der Waals radii of 2.88 Å), with the phenolic group of mol­ecule *A* related by *a*-glide symmetry. These two distinct hydrogen-bond inter­actions can be clearly detected in the IR spectrum of **1** with strong OH vibrational frequencies of 3409 and 3527 cm^−1^, respectively. The result is the formation of an infinite chain of mol­ecules alternately linked by O—H⋯O and O—H⋯π inter­actions that propagates along the *a* axis of the crystal (Fig. 5 ▸).

## Database survey   

A search of the Cambridge Structural Database (CSD, Version 5.38, update May 2017; Groom *et al.*, 2016 ▸) for the *thia*-Dianin framework, **2**, yielded 14 hits, all of which were genuine examples of analogues of the material under investigation. Although there are no entries for the empty racemic *R* 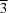 host of *thia*-Dianin’s compound, there are eight entries for the following host–guest clathrates: ethanol (CSD refcode HPTHCR; MacNicol *et al.*, 1969 ▸), 2,5,5-tri­methyl­hex-3-yn-2-ol (TCHHXO; MacNicol & Wilson, 1971 ▸), cyclo­pentane and cyclo­octane (METCCP and MSOCYO10, repectively; Hardy *et al.*, 1979 ▸), and propan-2-ol at four different temperatures demonstrating three commensurate phase changes in the host lattice (VANFOI, 371 K, VANFOI01, 295 K, VANFOI02, 200 K, and VANFUO, 90 K; Frampton *et al.*, 2017).

*Thia*-Dianin’s compound, **2**, was also found in the 1:1 quasi-racemic *R*3 host with Dianin’s compound, **3**, in the following three entries: apohost (BIBNAD and BIBNAD01) and CCl_4_/H_2_O (HIDQAO) (Frampton *et al.*, 2013 ▸).

The structure and absolute stereochemistry determination of the resolved *S*-enantio­mer of *thia*-Dianin’s compound was used in the formation of the quasi-racemates above (BIBNEH; Frampton *et al.*, 2013 ▸).

Two further examples demonstrating a slightly modified framework include the 7-methyl analogue (HPMTCM; Hardy *et al.*, 1977 ▸) and the oxidized sulfone, **4** (KUTDUY; Frampton *et al.*, 1992 ▸).

## Synthesis and crystallization   

Compound **1** was produced, as described in the literature, by the action of gaseous hydrogen chloride on a mixture of phenol and 4-methyl-4-(1-naphthyl­sulfanyl)pentan-2-one (Hardy *et al.*, 1979 ▸). Unsolvated colourless prisms suitable for X-ray diffraction were obtained by recrystallisation from nitro­methane solution, m.p. 425–427 K.

## Refinement   

The positional coordinates of the O-bound H atom were located from a difference Fourier map and freely refined along with an isotropic displacement parameter. All the remaining H atoms were placed geometrically in idealized positions and refined using a riding model (including free rotation about the methyl C—C bond), with C–H = 0.95–0.99 Å and *U*_iso_(H) = 1.5*U*_eq_(C) for methyl groups, or 1.2*U*_eq_(C) for other H atoms. Initial refinements demonstrated that the crystal was a near-perfect twin rotated 179° about the [001] direction. The refinement for the twinned data set (*R*_int_ = 0.0747) converged with *R*[*F* ^2^ > 2σ(*F* ^2^)], *wR*(*F* ^2^), *S* = 0.0611, 0.2328, 1.115, Flack *x* = 0.01 (4) (Flack, 1983 ▸) by classical fit to all intensities. Deconvolution of the twin yielded a data set that was 91.7% complete to 0.80 Å after the reflections where the overlap was greater than 0.8 were removed. Crystal data, data collection, and structure refinement details for the full data set with individual twin components are summarized in Table 2 ▸.

## Supplementary Material

Crystal structure: contains datablock(s) I, global. DOI: 10.1107/S2056989017014608/hb7711sup1.cif

Structure factors: contains datablock(s) I. DOI: 10.1107/S2056989017014608/hb7711Isup2.hkl

Supporting information file. DOI: 10.1107/S2056989017014608/hb7711Isup3.cml

CCDC reference: 1579039

Additional supporting information:  crystallographic information; 3D view; checkCIF report

## Figures and Tables

**Figure 2:**
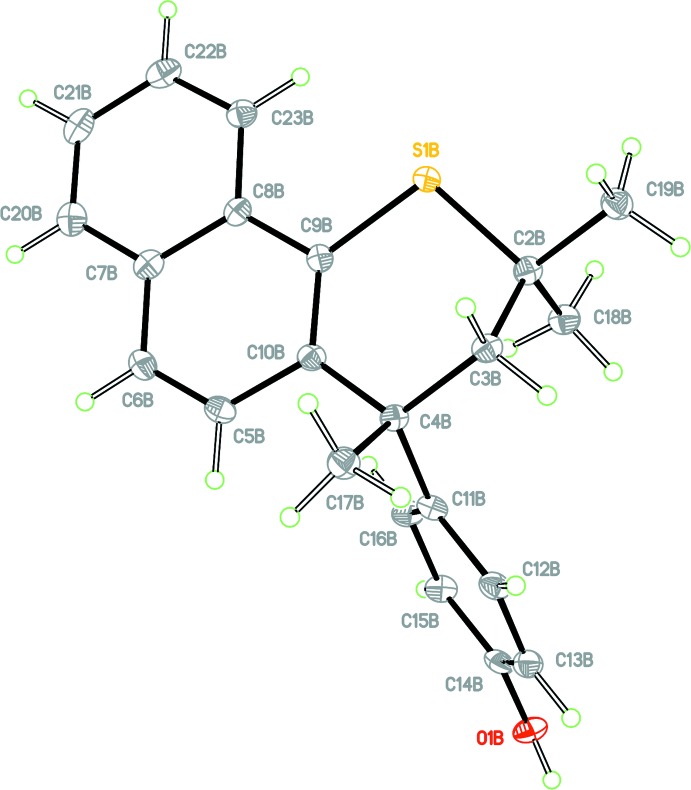
View of mol­ecule *B* of the asymmetric unit with the atom labelling. Displacement ellipsoids are drawn at the 50% probability level.

**Table 1:** Hydrogen-bond geometry (Å, °) *Cg*1 is the centroid of the C11*A*–C16*A* ring.

*D*—H⋯*A*	*D*—H	H⋯*A*	*D*⋯*A*	*D*—H⋯*A*
O1*A*—H1*A*⋯O1*B*	0.84 (6)	1.96 (6)	2.777 (4)	162 (6)
O1*B*—H1*B*⋯*Cg*1^i^	0.83 (6)	3.18 (6)	3.959 (4)	158 (6)

Symmetry code: (i) 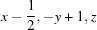.

**Table 2:** Experimental details

Crystal data	
Chemical formula	C_22_H_22_OS
*M*_r_	334.45
Crystal system, space group	Monoclinic, *I* *a*
Temperature (K)	100
*a*, *b*, *c* (Å)	10.3190 (3), 20.6009 (7), 15.8756 (5)
β (°)	91.640 (3)
*V* (Å^3^)	3373.5 (2)
*Z*	8
Radiation type	Cu *K*α
μ (mm^−1^)	1.72
Crystal size (mm)	0.36 × 0.14 × 0.05

Data collection	
Diffractometer	Rigaku Oxford Diffraction SuperNova, Dualflex, AtlasS2
Absorption correction	Analytical [*CrysAlis PRO* (Rigaku Oxford Diffraction, 2015 ▸), based on expressions derived by Clark & Reid (1995 ▸)]
*T*_min_, *T*_max_	0.740, 0.914
No. of measured, independent and observed [*I* > 2σ(*I*)] reflections	7560, 7560, 7158
(sin θ/λ)_max_ (Å^−1^)	0.625

Refinement	
*R*[*F* ^2^ > 2σ(*F* ^2^)], *wR*(*F* ^2^), *S*	0.037, 0.103, 1.02
No. of reflections	7560
No. of parameters	447
No. of restraints	2
H-atom treatment	H atoms treated by a mixture of independent and constrained refinement
Δρ_max_, Δρ_min_ (e Å^−3^)	0.29, −0.26
Absolute structure	Classical Flack method preferred over Parsons because s.u. lower. Value quoted is from the twinned data set
Absolute structure parameter	0.01 (4)

Computer programs: *CrysAlis PRO* (Rigaku Oxford Diffraction, 2015 ▸), *SHELXD2014/6* and *SHELXL2014/6* (Sheldrick, 2015 ▸), *SHELXTL* (Sheldrick, 2008 ▸), *Mercury* (Macrae *et al.*, 2008 ▸) and *publCIF* (Westrip, 2010 ▸).

## supplementary crystallographic information

### Crystal data

**Table:**

C_22_H_22_OS	*F*(000) = 1424
*M**_r_* = 334.45	*D*_x_ = 1.317 Mg m^−^^3^
Monoclinic, *I**a*	Cu *K*α radiation, λ = 1.54184 Å
*a* = 10.3190 (3) Å	Cell parameters from 5117 reflections
*b* = 20.6009 (7) Å	θ = 3.5–76.6°
*c* = 15.8756 (5) Å	µ = 1.72 mm^−^^1^
β = 91.640 (3)°	*T* = 100 K
*V* = 3373.5 (2) Å^3^	Plate, colourless
*Z* = 8	0.36 × 0.14 × 0.05 mm

### Data collection

**Table:**

Rigaku Oxford Diffraction SuperNova, Dualflex, AtlasS2 diffractometer	7560 independent reflections
Radiation source: fine-focus sealed X-ray tube, Enhance (Cu) X-ray source	7158 reflections with *I* > 2σ(*I*)
Detector resolution: 5.2921 pixels mm^-1^	θ_max_ = 74.5°, θ_min_ = 3.5°
ω scans	*h* = −12→12
Absorption correction: analytical [CrysAlis PRO (Rigaku Oxford Diffraction, 2015), based on expressions derived by Clark & Reid (1995)]	*k* = −25→25
*T*_min_ = 0.740, *T*_max_ = 0.914	*l* = −19→19
7560 measured reflections	

### Refinement

**Table:**

Refinement on *F*^2^	Hydrogen site location: mixed
Least-squares matrix: full	H atoms treated by a mixture of independent and constrained refinement
*R*[*F*^2^ > 2σ(*F*^2^)] = 0.037	*w* = 1/[σ^2^(*F*_o_^2^) + (0.0825*P*)^2^] where *P* = (*F*_o_^2^ + 2*F*_c_^2^)/3
*wR*(*F*^2^) = 0.103	(Δ/σ)_max_ < 0.001
*S* = 1.02	Δρ_max_ = 0.29 e Å^−^^3^
7560 reflections	Δρ_min_ = −0.26 e Å^−^^3^
447 parameters	Absolute structure: Classical Flack method preferred over Parsons because s.u. lower. Value quoted is from the twinned data set
2 restraints	Absolute structure parameter: 0.01 (4)
Primary atom site location: structure-invariant direct methods	

### Special details

**Table:**

Geometry. All esds (except the esd in the dihedral angle between two l.s. planes) are estimated using the full covariance matrix. The cell esds are taken into account individually in the estimation of esds in distances, angles and torsion angles; correlations between esds in cell parameters are only used when they are defined by crystal symmetry. An approximate (isotropic) treatment of cell esds is used for estimating esds involving l.s. planes.
Refinement. Refined as a 2-component perfect twin

### Fractional atomic coordinates and isotropic or equivalent isotropic displacement parameters (Å^2^)

**Table:**

	*x*	*y*	*z*	*U*_iso_*/*U*_eq_	
S1A	0.51009 (7)	0.35123 (4)	0.09817 (5)	0.01486 (18)	
O1A	0.0378 (2)	0.43051 (14)	0.46900 (15)	0.0178 (5)	
H1A	0.052 (6)	0.470 (3)	0.477 (4)	0.049 (18)*	
C2A	0.3492 (3)	0.32341 (17)	0.1298 (2)	0.0147 (6)	
C3A	0.3674 (3)	0.27563 (16)	0.20243 (19)	0.0135 (6)	
H3AA	0.4203	0.2390	0.1822	0.016*	
H3AB	0.2811	0.2579	0.2154	0.016*	
C4A	0.4307 (3)	0.30021 (16)	0.28593 (19)	0.0121 (6)	
C5A	0.6335 (3)	0.35373 (16)	0.3449 (2)	0.0148 (6)	
H5AA	0.6058	0.3391	0.3982	0.018*	
C6A	0.7480 (3)	0.38594 (17)	0.3417 (2)	0.0153 (7)	
H6AA	0.7983	0.3931	0.3919	0.018*	
C7A	0.7925 (3)	0.40907 (16)	0.2631 (2)	0.0142 (6)	
C8A	0.7129 (3)	0.39895 (16)	0.1893 (2)	0.0135 (6)	
C9A	0.5928 (3)	0.36405 (16)	0.1954 (2)	0.0124 (6)	
C10A	0.5526 (3)	0.34068 (16)	0.2725 (2)	0.0124 (6)	
C11A	0.3310 (3)	0.33736 (16)	0.33830 (19)	0.0123 (6)	
C12A	0.2219 (3)	0.30484 (16)	0.36687 (19)	0.0148 (6)	
H12A	0.2126	0.2598	0.3555	0.018*	
C13A	0.1268 (3)	0.33628 (17)	0.4112 (2)	0.0149 (6)	
H13A	0.0541	0.3127	0.4302	0.018*	
C14A	0.1377 (3)	0.40280 (17)	0.42807 (18)	0.0133 (6)	
C15A	0.2463 (3)	0.43601 (17)	0.40170 (19)	0.0141 (6)	
H15A	0.2561	0.4809	0.4140	0.017*	
C16A	0.3413 (3)	0.40343 (16)	0.35715 (19)	0.0126 (6)	
H16A	0.4149	0.4268	0.3392	0.015*	
C17A	0.4708 (3)	0.23763 (17)	0.3346 (2)	0.0158 (6)	
H17A	0.5427	0.2166	0.3062	0.024*	
H17B	0.3968	0.2079	0.3359	0.024*	
H17C	0.4982	0.2490	0.3924	0.024*	
C18A	0.2637 (3)	0.38209 (17)	0.1493 (2)	0.0171 (7)	
H18A	0.1811	0.3670	0.1712	0.026*	
H18B	0.2474	0.4072	0.0977	0.026*	
H18C	0.3079	0.4095	0.1916	0.026*	
C19A	0.2932 (4)	0.28720 (18)	0.0526 (2)	0.0176 (7)	
H19A	0.2037	0.2742	0.0631	0.026*	
H19B	0.3456	0.2485	0.0422	0.026*	
H19C	0.2943	0.3158	0.0033	0.026*	
C20A	0.9137 (4)	0.44096 (18)	0.2562 (2)	0.0190 (7)	
H20A	0.9663	0.4478	0.3055	0.023*	
C21A	0.9561 (4)	0.46198 (18)	0.1802 (2)	0.0206 (7)	
H21A	1.0380	0.4827	0.1765	0.025*	
C22A	0.8771 (4)	0.45259 (19)	0.1076 (2)	0.0208 (7)	
H22A	0.9064	0.4672	0.0546	0.025*	
C23A	0.7588 (3)	0.42281 (18)	0.1118 (2)	0.0167 (7)	
H23A	0.7065	0.4180	0.0619	0.020*	
S1B	0.52638 (7)	0.64688 (4)	0.89907 (5)	0.01474 (18)	
O1B	0.0356 (3)	0.56057 (13)	0.51496 (15)	0.0181 (5)	
H1B	−0.014 (6)	0.585 (3)	0.488 (4)	0.047 (16)*	
C2B	0.3602 (3)	0.67165 (16)	0.8687 (2)	0.0138 (6)	
C3B	0.3668 (3)	0.72023 (16)	0.79605 (19)	0.0135 (6)	
H3BA	0.4195	0.7577	0.8160	0.016*	
H3BB	0.2779	0.7365	0.7842	0.016*	
C4B	0.4223 (3)	0.69694 (16)	0.71187 (19)	0.0124 (6)	
C5B	0.6165 (4)	0.64319 (16)	0.6496 (2)	0.0152 (6)	
H5BA	0.5798	0.6559	0.5966	0.018*	
C6B	0.7329 (4)	0.61284 (18)	0.6515 (2)	0.0163 (7)	
H6BA	0.7765	0.6058	0.6004	0.020*	
C7B	0.7904 (3)	0.59146 (16)	0.7290 (2)	0.0148 (6)	
C8B	0.7207 (3)	0.60154 (16)	0.8042 (2)	0.0129 (6)	
C9B	0.5974 (3)	0.63472 (16)	0.8002 (2)	0.0124 (6)	
C10B	0.5467 (3)	0.65688 (16)	0.7238 (2)	0.0131 (6)	
C11B	0.3193 (3)	0.66021 (17)	0.65836 (19)	0.0136 (6)	
C12B	0.2095 (3)	0.69261 (16)	0.62686 (19)	0.0148 (6)	
H12B	0.1993	0.7375	0.6388	0.018*	
C13B	0.1148 (3)	0.66103 (18)	0.5785 (2)	0.0162 (7)	
H13B	0.0409	0.6841	0.5577	0.019*	
C14B	0.1287 (3)	0.59546 (17)	0.56087 (19)	0.0151 (6)	
C15B	0.2358 (3)	0.56191 (17)	0.5911 (2)	0.0158 (6)	
H15B	0.2451	0.5170	0.5792	0.019*	
C16B	0.3308 (3)	0.59444 (17)	0.6394 (2)	0.0162 (7)	
H16B	0.4048	0.5712	0.6598	0.019*	
C17B	0.4583 (3)	0.76053 (18)	0.6652 (2)	0.0164 (6)	
H17D	0.4825	0.7502	0.6075	0.025*	
H17E	0.5315	0.7815	0.6950	0.025*	
H17F	0.3836	0.7899	0.6638	0.025*	
C18B	0.2769 (3)	0.61185 (17)	0.8491 (2)	0.0171 (7)	
H18D	0.1910	0.6256	0.8282	0.026*	
H18E	0.2679	0.5861	0.9006	0.026*	
H18F	0.3184	0.5855	0.8062	0.026*	
C19B	0.3099 (4)	0.70637 (18)	0.9469 (2)	0.0173 (6)	
H19D	0.2174	0.7159	0.9385	0.026*	
H19E	0.3577	0.7470	0.9559	0.026*	
H19F	0.3223	0.6783	0.9964	0.026*	
C20B	0.9126 (4)	0.56092 (18)	0.7331 (2)	0.0179 (7)	
H20B	0.9581	0.5542	0.6826	0.021*	
C21B	0.9672 (4)	0.54077 (17)	0.8083 (2)	0.0187 (7)	
H21B	1.0499	0.5204	0.8100	0.022*	
C22B	0.8995 (4)	0.55050 (17)	0.8832 (2)	0.0186 (7)	
H22B	0.9374	0.5371	0.9356	0.022*	
C23B	0.7789 (3)	0.57927 (17)	0.8812 (2)	0.0158 (6)	
H23B	0.7338	0.5843	0.9321	0.019*	

### Atomic displacement parameters (Å^2^)

**Table:**

	*U*^11^	*U*^22^	*U*^33^	*U*^12^	*U*^13^	*U*^23^
S1A	0.0142 (4)	0.0190 (4)	0.0113 (4)	−0.0012 (3)	−0.0001 (3)	0.0011 (3)
O1A	0.0157 (12)	0.0184 (13)	0.0196 (11)	−0.0003 (10)	0.0041 (9)	−0.0050 (9)
C2A	0.0134 (15)	0.0158 (17)	0.0149 (15)	−0.0004 (13)	0.0000 (11)	0.0002 (12)
C3A	0.0138 (16)	0.0114 (16)	0.0152 (14)	0.0009 (12)	0.0008 (11)	−0.0025 (11)
C4A	0.0130 (15)	0.0099 (15)	0.0134 (14)	−0.0002 (12)	0.0010 (11)	0.0003 (10)
C5A	0.0170 (17)	0.0138 (16)	0.0137 (15)	0.0024 (12)	0.0018 (12)	−0.0003 (11)
C6A	0.0170 (17)	0.0131 (17)	0.0157 (16)	0.0009 (13)	−0.0021 (12)	−0.0030 (12)
C7A	0.0131 (16)	0.0099 (16)	0.0194 (16)	0.0026 (13)	0.0009 (12)	−0.0009 (11)
C8A	0.0139 (16)	0.0099 (16)	0.0167 (16)	0.0020 (13)	0.0024 (11)	−0.0008 (11)
C9A	0.0115 (16)	0.0116 (15)	0.0140 (14)	0.0030 (12)	−0.0008 (11)	−0.0005 (11)
C10A	0.0143 (17)	0.0105 (15)	0.0126 (15)	0.0016 (13)	0.0011 (11)	−0.0016 (11)
C11A	0.0149 (16)	0.0124 (15)	0.0095 (13)	−0.0006 (13)	−0.0011 (11)	0.0009 (11)
C12A	0.0180 (16)	0.0137 (16)	0.0127 (14)	−0.0023 (13)	0.0009 (11)	−0.0015 (11)
C13A	0.0148 (16)	0.0153 (16)	0.0145 (15)	−0.0022 (13)	0.0004 (11)	−0.0009 (12)
C14A	0.0138 (15)	0.0166 (16)	0.0095 (14)	0.0013 (12)	−0.0007 (10)	−0.0010 (11)
C15A	0.0192 (17)	0.0108 (15)	0.0122 (14)	−0.0007 (12)	−0.0005 (12)	−0.0012 (11)
C16A	0.0122 (15)	0.0125 (16)	0.0129 (15)	−0.0028 (12)	−0.0012 (11)	0.0022 (11)
C17A	0.0177 (16)	0.0120 (16)	0.0175 (15)	−0.0006 (12)	−0.0023 (12)	0.0014 (11)
C18A	0.0178 (17)	0.0154 (17)	0.0180 (16)	0.0034 (13)	−0.0005 (12)	0.0014 (12)
C19A	0.0200 (17)	0.0190 (18)	0.0135 (15)	−0.0002 (13)	−0.0013 (12)	−0.0008 (12)
C20A	0.0170 (17)	0.0149 (18)	0.0251 (17)	−0.0011 (14)	−0.0003 (12)	−0.0020 (12)
C21A	0.0165 (17)	0.0136 (17)	0.032 (2)	−0.0022 (13)	0.0031 (13)	0.0003 (13)
C22A	0.0212 (18)	0.0188 (18)	0.0227 (17)	−0.0032 (14)	0.0071 (13)	0.0024 (13)
C23A	0.0172 (16)	0.0142 (17)	0.0188 (16)	−0.0004 (13)	0.0023 (12)	−0.0001 (12)
S1B	0.0136 (4)	0.0194 (4)	0.0112 (4)	0.0009 (3)	−0.0001 (3)	0.0008 (3)
O1B	0.0176 (13)	0.0176 (13)	0.0187 (12)	0.0001 (10)	−0.0071 (9)	−0.0017 (9)
C2B	0.0143 (15)	0.0122 (16)	0.0148 (14)	0.0010 (12)	0.0000 (11)	−0.0007 (11)
C3B	0.0147 (16)	0.0108 (16)	0.0150 (15)	0.0002 (12)	−0.0015 (11)	−0.0026 (11)
C4B	0.0134 (15)	0.0111 (16)	0.0125 (14)	0.0012 (12)	−0.0007 (11)	−0.0008 (11)
C5B	0.0192 (18)	0.0140 (16)	0.0122 (15)	−0.0001 (13)	−0.0002 (12)	−0.0012 (11)
C6B	0.0179 (17)	0.0166 (17)	0.0145 (15)	−0.0006 (13)	0.0031 (12)	−0.0013 (12)
C7B	0.0154 (17)	0.0098 (16)	0.0191 (16)	−0.0006 (13)	−0.0014 (12)	−0.0017 (11)
C8B	0.0129 (16)	0.0087 (15)	0.0170 (16)	−0.0027 (12)	−0.0001 (11)	0.0005 (11)
C9B	0.0131 (16)	0.0104 (15)	0.0136 (14)	−0.0011 (12)	0.0011 (11)	−0.0009 (11)
C10B	0.0144 (17)	0.0122 (16)	0.0127 (15)	−0.0001 (13)	−0.0006 (12)	−0.0029 (11)
C11B	0.0178 (17)	0.0127 (15)	0.0101 (14)	−0.0006 (13)	−0.0006 (11)	0.0002 (11)
C12B	0.0190 (17)	0.0137 (16)	0.0117 (14)	0.0022 (13)	0.0004 (11)	−0.0020 (11)
C13B	0.0171 (17)	0.0176 (17)	0.0138 (15)	0.0032 (13)	−0.0001 (12)	0.0000 (12)
C14B	0.0185 (16)	0.0180 (17)	0.0087 (14)	−0.0039 (13)	0.0002 (11)	−0.0012 (11)
C15B	0.0201 (17)	0.0115 (16)	0.0156 (15)	−0.0005 (13)	−0.0021 (12)	−0.0007 (11)
C16B	0.0186 (17)	0.0145 (17)	0.0152 (15)	0.0019 (13)	−0.0026 (12)	0.0011 (12)
C17B	0.0171 (16)	0.0158 (17)	0.0164 (15)	0.0009 (13)	0.0004 (11)	0.0005 (12)
C18B	0.0175 (17)	0.0158 (18)	0.0178 (16)	−0.0026 (13)	−0.0007 (12)	0.0010 (12)
C19B	0.0184 (16)	0.0175 (17)	0.0159 (15)	0.0005 (13)	0.0008 (11)	−0.0013 (12)
C20B	0.0189 (18)	0.0123 (17)	0.0226 (17)	−0.0023 (13)	0.0025 (13)	−0.0017 (12)
C21B	0.0143 (17)	0.0122 (17)	0.0296 (18)	−0.0001 (12)	−0.0006 (13)	0.0004 (13)
C22B	0.0192 (18)	0.0136 (17)	0.0226 (17)	−0.0014 (13)	−0.0056 (13)	0.0039 (13)
C23B	0.0164 (17)	0.0141 (16)	0.0166 (16)	−0.0020 (13)	−0.0012 (12)	0.0002 (11)

### Geometric parameters (Å, º)

**Table:**

S1A—C9A	1.762 (3)	S1B—C9B	1.768 (3)
S1A—C2A	1.839 (4)	S1B—C2B	1.840 (3)
O1A—C14A	1.360 (4)	O1B—C14B	1.389 (4)
O1A—H1A	0.84 (6)	O1B—H1B	0.83 (6)
C2A—C3A	1.524 (4)	C2B—C18B	1.529 (5)
C2A—C19A	1.533 (4)	C2B—C3B	1.530 (4)
C2A—C18A	1.534 (5)	C2B—C19B	1.537 (4)
C3A—C4A	1.546 (4)	C3B—C4B	1.545 (4)
C3A—H3AA	0.9900	C3B—H3BA	0.9900
C3A—H3AB	0.9900	C3B—H3BB	0.9900
C4A—C10A	1.529 (5)	C4B—C10B	1.533 (5)
C4A—C11A	1.544 (4)	C4B—C11B	1.540 (4)
C4A—C17A	1.553 (4)	C4B—C17B	1.555 (5)
C5A—C6A	1.357 (5)	C5B—C6B	1.353 (5)
C5A—C10A	1.427 (5)	C5B—C10B	1.427 (4)
C5A—H5AA	0.9500	C5B—H5BA	0.9500
C6A—C7A	1.424 (5)	C6B—C7B	1.421 (5)
C6A—H6AA	0.9500	C6B—H6BA	0.9500
C7A—C20A	1.419 (5)	C7B—C20B	1.409 (5)
C7A—C8A	1.427 (4)	C7B—C8B	1.426 (5)
C8A—C23A	1.419 (5)	C8B—C23B	1.423 (5)
C8A—C9A	1.439 (5)	C8B—C9B	1.444 (5)
C9A—C10A	1.390 (5)	C9B—C10B	1.385 (5)
C11A—C16A	1.397 (5)	C11B—C16B	1.394 (5)
C11A—C12A	1.397 (5)	C11B—C12B	1.396 (5)
C12A—C13A	1.384 (5)	C12B—C13B	1.387 (5)
C12A—H12A	0.9500	C12B—H12B	0.9500
C13A—C14A	1.400 (5)	C13B—C14B	1.388 (5)
C13A—H13A	0.9500	C13B—H13B	0.9500
C14A—C15A	1.388 (5)	C14B—C15B	1.379 (5)
C15A—C16A	1.397 (5)	C15B—C16B	1.398 (5)
C15A—H15A	0.9500	C15B—H15B	0.9500
C16A—H16A	0.9500	C16B—H16B	0.9500
C17A—H17A	0.9800	C17B—H17D	0.9800
C17A—H17B	0.9800	C17B—H17E	0.9800
C17A—H17C	0.9800	C17B—H17F	0.9800
C18A—H18A	0.9800	C18B—H18D	0.9800
C18A—H18B	0.9800	C18B—H18E	0.9800
C18A—H18C	0.9800	C18B—H18F	0.9800
C19A—H19A	0.9800	C19B—H19D	0.9800
C19A—H19B	0.9800	C19B—H19E	0.9800
C19A—H19C	0.9800	C19B—H19F	0.9800
C20A—C21A	1.365 (5)	C20B—C21B	1.370 (5)
C20A—H20A	0.9500	C20B—H20B	0.9500
C21A—C22A	1.407 (5)	C21B—C22B	1.411 (5)
C21A—H21A	0.9500	C21B—H21B	0.9500
C22A—C23A	1.370 (5)	C22B—C23B	1.378 (5)
C22A—H22A	0.9500	C22B—H22B	0.9500
C23A—H23A	0.9500	C23B—H23B	0.9500
			
C9A—S1A—C2A	103.04 (15)	C9B—S1B—C2B	102.31 (15)
C14A—O1A—H1A	111 (4)	C14B—O1B—H1B	111 (4)
C3A—C2A—C19A	109.0 (3)	C18B—C2B—C3B	114.3 (3)
C3A—C2A—C18A	114.5 (3)	C18B—C2B—C19B	109.7 (3)
C19A—C2A—C18A	109.8 (3)	C3B—C2B—C19B	109.2 (3)
C3A—C2A—S1A	108.4 (2)	C18B—C2B—S1B	110.1 (2)
C19A—C2A—S1A	104.8 (2)	C3B—C2B—S1B	108.5 (2)
C18A—C2A—S1A	109.8 (2)	C19B—C2B—S1B	104.6 (2)
C2A—C3A—C4A	118.5 (3)	C2B—C3B—C4B	118.3 (3)
C2A—C3A—H3AA	107.7	C2B—C3B—H3BA	107.7
C4A—C3A—H3AA	107.7	C4B—C3B—H3BA	107.7
C2A—C3A—H3AB	107.7	C2B—C3B—H3BB	107.7
C4A—C3A—H3AB	107.7	C4B—C3B—H3BB	107.7
H3AA—C3A—H3AB	107.1	H3BA—C3B—H3BB	107.1
C10A—C4A—C11A	111.6 (3)	C10B—C4B—C11B	111.5 (3)
C10A—C4A—C3A	112.8 (3)	C10B—C4B—C3B	112.9 (3)
C11A—C4A—C3A	110.6 (3)	C11B—C4B—C3B	111.3 (3)
C10A—C4A—C17A	108.3 (3)	C10B—C4B—C17B	107.6 (3)
C11A—C4A—C17A	108.4 (3)	C11B—C4B—C17B	108.8 (3)
C3A—C4A—C17A	104.8 (3)	C3B—C4B—C17B	104.5 (3)
C6A—C5A—C10A	123.5 (3)	C6B—C5B—C10B	122.8 (3)
C6A—C5A—H5AA	118.2	C6B—C5B—H5BA	118.6
C10A—C5A—H5AA	118.2	C10B—C5B—H5BA	118.6
C5A—C6A—C7A	120.0 (3)	C5B—C6B—C7B	120.7 (3)
C5A—C6A—H6AA	120.0	C5B—C6B—H6BA	119.7
C7A—C6A—H6AA	120.0	C7B—C6B—H6BA	119.7
C20A—C7A—C6A	122.0 (3)	C20B—C7B—C6B	122.0 (3)
C20A—C7A—C8A	119.5 (3)	C20B—C7B—C8B	119.9 (3)
C6A—C7A—C8A	118.5 (3)	C6B—C7B—C8B	118.1 (3)
C23A—C8A—C7A	117.6 (3)	C23B—C8B—C7B	117.5 (3)
C23A—C8A—C9A	122.8 (3)	C23B—C8B—C9B	122.6 (3)
C7A—C8A—C9A	119.6 (3)	C7B—C8B—C9B	119.9 (3)
C10A—C9A—C8A	120.8 (3)	C10B—C9B—C8B	120.3 (3)
C10A—C9A—S1A	124.8 (3)	C10B—C9B—S1B	125.1 (3)
C8A—C9A—S1A	114.4 (2)	C8B—C9B—S1B	114.6 (2)
C9A—C10A—C5A	117.5 (3)	C9B—C10B—C5B	118.1 (3)
C9A—C10A—C4A	125.4 (3)	C9B—C10B—C4B	125.5 (3)
C5A—C10A—C4A	117.1 (3)	C5B—C10B—C4B	116.4 (3)
C16A—C11A—C12A	117.0 (3)	C16B—C11B—C12B	117.4 (3)
C16A—C11A—C4A	123.4 (3)	C16B—C11B—C4B	122.3 (3)
C12A—C11A—C4A	119.5 (3)	C12B—C11B—C4B	120.2 (3)
C13A—C12A—C11A	122.0 (3)	C13B—C12B—C11B	121.7 (3)
C13A—C12A—H12A	119.0	C13B—C12B—H12B	119.1
C11A—C12A—H12A	119.0	C11B—C12B—H12B	119.1
C12A—C13A—C14A	120.1 (3)	C12B—C13B—C14B	119.5 (3)
C12A—C13A—H13A	120.0	C12B—C13B—H13B	120.2
C14A—C13A—H13A	120.0	C14B—C13B—H13B	120.2
O1A—C14A—C15A	124.5 (3)	C15B—C14B—C13B	120.3 (3)
O1A—C14A—C13A	116.4 (3)	C15B—C14B—O1B	117.3 (3)
C15A—C14A—C13A	119.1 (3)	C13B—C14B—O1B	122.4 (3)
C14A—C15A—C16A	120.0 (3)	C14B—C15B—C16B	119.6 (3)
C14A—C15A—H15A	120.0	C14B—C15B—H15B	120.2
C16A—C15A—H15A	120.0	C16B—C15B—H15B	120.2
C15A—C16A—C11A	121.8 (3)	C11B—C16B—C15B	121.4 (3)
C15A—C16A—H16A	119.1	C11B—C16B—H16B	119.3
C11A—C16A—H16A	119.1	C15B—C16B—H16B	119.3
C4A—C17A—H17A	109.5	C4B—C17B—H17D	109.5
C4A—C17A—H17B	109.5	C4B—C17B—H17E	109.5
H17A—C17A—H17B	109.5	H17D—C17B—H17E	109.5
C4A—C17A—H17C	109.5	C4B—C17B—H17F	109.5
H17A—C17A—H17C	109.5	H17D—C17B—H17F	109.5
H17B—C17A—H17C	109.5	H17E—C17B—H17F	109.5
C2A—C18A—H18A	109.5	C2B—C18B—H18D	109.5
C2A—C18A—H18B	109.5	C2B—C18B—H18E	109.5
H18A—C18A—H18B	109.5	H18D—C18B—H18E	109.5
C2A—C18A—H18C	109.5	C2B—C18B—H18F	109.5
H18A—C18A—H18C	109.5	H18D—C18B—H18F	109.5
H18B—C18A—H18C	109.5	H18E—C18B—H18F	109.5
C2A—C19A—H19A	109.5	C2B—C19B—H19D	109.5
C2A—C19A—H19B	109.5	C2B—C19B—H19E	109.5
H19A—C19A—H19B	109.5	H19D—C19B—H19E	109.5
C2A—C19A—H19C	109.5	C2B—C19B—H19F	109.5
H19A—C19A—H19C	109.5	H19D—C19B—H19F	109.5
H19B—C19A—H19C	109.5	H19E—C19B—H19F	109.5
C21A—C20A—C7A	121.4 (3)	C21B—C20B—C7B	121.4 (3)
C21A—C20A—H20A	119.3	C21B—C20B—H20B	119.3
C7A—C20A—H20A	119.3	C7B—C20B—H20B	119.3
C20A—C21A—C22A	119.2 (4)	C20B—C21B—C22B	119.4 (4)
C20A—C21A—H21A	120.4	C20B—C21B—H21B	120.3
C22A—C21A—H21A	120.4	C22B—C21B—H21B	120.3
C23A—C22A—C21A	121.2 (3)	C23B—C22B—C21B	120.7 (3)
C23A—C22A—H22A	119.4	C23B—C22B—H22B	119.7
C21A—C22A—H22A	119.4	C21B—C22B—H22B	119.7
C22A—C23A—C8A	121.2 (3)	C22B—C23B—C8B	121.1 (3)
C22A—C23A—H23A	119.4	C22B—C23B—H23B	119.4
C8A—C23A—H23A	119.4	C8B—C23B—H23B	119.4
			
C9A—S1A—C2A—C3A	−42.3 (2)	C9B—S1B—C2B—C18B	82.3 (2)
C9A—S1A—C2A—C19A	−158.7 (2)	C9B—S1B—C2B—C3B	−43.5 (2)
C9A—S1A—C2A—C18A	83.4 (2)	C9B—S1B—C2B—C19B	−159.9 (2)
C19A—C2A—C3A—C4A	176.4 (3)	C18B—C2B—C3B—C4B	−60.1 (4)
C18A—C2A—C3A—C4A	−60.1 (4)	C19B—C2B—C3B—C4B	176.7 (3)
S1A—C2A—C3A—C4A	62.8 (3)	S1B—C2B—C3B—C4B	63.3 (3)
C2A—C3A—C4A—C10A	−46.3 (4)	C2B—C3B—C4B—C10B	−44.8 (4)
C2A—C3A—C4A—C11A	79.5 (4)	C2B—C3B—C4B—C11B	81.4 (4)
C2A—C3A—C4A—C17A	−163.9 (3)	C2B—C3B—C4B—C17B	−161.4 (3)
C10A—C5A—C6A—C7A	0.4 (5)	C10B—C5B—C6B—C7B	1.5 (6)
C5A—C6A—C7A—C20A	−177.7 (3)	C5B—C6B—C7B—C20B	−178.7 (3)
C5A—C6A—C7A—C8A	1.6 (5)	C5B—C6B—C7B—C8B	1.4 (5)
C20A—C7A—C8A—C23A	−1.1 (5)	C20B—C7B—C8B—C23B	−0.5 (5)
C6A—C7A—C8A—C23A	179.6 (3)	C6B—C7B—C8B—C23B	179.4 (3)
C20A—C7A—C8A—C9A	177.2 (3)	C20B—C7B—C8B—C9B	178.0 (3)
C6A—C7A—C8A—C9A	−2.1 (5)	C6B—C7B—C8B—C9B	−2.1 (5)
C23A—C8A—C9A—C10A	179.0 (3)	C23B—C8B—C9B—C10B	178.3 (3)
C7A—C8A—C9A—C10A	0.7 (5)	C7B—C8B—C9B—C10B	−0.1 (5)
C23A—C8A—C9A—S1A	1.9 (4)	C23B—C8B—C9B—S1B	1.3 (4)
C7A—C8A—C9A—S1A	−176.4 (2)	C7B—C8B—C9B—S1B	−177.2 (2)
C2A—S1A—C9A—C10A	15.4 (3)	C2B—S1B—C9B—C10B	15.7 (3)
C2A—S1A—C9A—C8A	−167.7 (2)	C2B—S1B—C9B—C8B	−167.4 (2)
C8A—C9A—C10A—C5A	1.1 (5)	C8B—C9B—C10B—C5B	3.0 (5)
S1A—C9A—C10A—C5A	177.9 (2)	S1B—C9B—C10B—C5B	179.7 (3)
C8A—C9A—C10A—C4A	−176.8 (3)	C8B—C9B—C10B—C4B	−175.3 (3)
S1A—C9A—C10A—C4A	0.0 (5)	S1B—C9B—C10B—C4B	1.4 (5)
C6A—C5A—C10A—C9A	−1.8 (5)	C6B—C5B—C10B—C9B	−3.8 (5)
C6A—C5A—C10A—C4A	176.3 (3)	C6B—C5B—C10B—C4B	174.7 (3)
C11A—C4A—C10A—C9A	−114.1 (4)	C11B—C4B—C10B—C9B	−117.2 (4)
C3A—C4A—C10A—C9A	11.2 (5)	C3B—C4B—C10B—C9B	8.9 (5)
C17A—C4A—C10A—C9A	126.6 (3)	C17B—C4B—C10B—C9B	123.7 (3)
C11A—C4A—C10A—C5A	67.9 (4)	C11B—C4B—C10B—C5B	64.5 (4)
C3A—C4A—C10A—C5A	−166.8 (3)	C3B—C4B—C10B—C5B	−169.4 (3)
C17A—C4A—C10A—C5A	−51.3 (4)	C17B—C4B—C10B—C5B	−54.6 (4)
C10A—C4A—C11A—C16A	11.0 (4)	C10B—C4B—C11B—C16B	12.2 (4)
C3A—C4A—C11A—C16A	−115.5 (3)	C3B—C4B—C11B—C16B	−114.8 (3)
C17A—C4A—C11A—C16A	130.2 (3)	C17B—C4B—C11B—C16B	130.7 (3)
C10A—C4A—C11A—C12A	−171.1 (3)	C10B—C4B—C11B—C12B	−167.8 (3)
C3A—C4A—C11A—C12A	62.4 (4)	C3B—C4B—C11B—C12B	65.2 (4)
C17A—C4A—C11A—C12A	−51.9 (4)	C17B—C4B—C11B—C12B	−49.4 (4)
C16A—C11A—C12A—C13A	0.6 (5)	C16B—C11B—C12B—C13B	0.0 (5)
C4A—C11A—C12A—C13A	−177.4 (3)	C4B—C11B—C12B—C13B	−179.9 (3)
C11A—C12A—C13A—C14A	0.5 (5)	C11B—C12B—C13B—C14B	0.1 (5)
C12A—C13A—C14A—O1A	177.5 (3)	C12B—C13B—C14B—C15B	0.0 (5)
C12A—C13A—C14A—C15A	−1.7 (5)	C12B—C13B—C14B—O1B	178.1 (3)
O1A—C14A—C15A—C16A	−177.5 (3)	C13B—C14B—C15B—C16B	−0.3 (5)
C13A—C14A—C15A—C16A	1.6 (5)	O1B—C14B—C15B—C16B	−178.4 (3)
C14A—C15A—C16A—C11A	−0.4 (5)	C12B—C11B—C16B—C15B	−0.3 (5)
C12A—C11A—C16A—C15A	−0.7 (5)	C4B—C11B—C16B—C15B	179.6 (3)
C4A—C11A—C16A—C15A	177.2 (3)	C14B—C15B—C16B—C11B	0.4 (5)
C6A—C7A—C20A—C21A	179.0 (3)	C6B—C7B—C20B—C21B	179.7 (3)
C8A—C7A—C20A—C21A	−0.3 (5)	C8B—C7B—C20B—C21B	−0.4 (5)
C7A—C20A—C21A—C22A	0.9 (5)	C7B—C20B—C21B—C22B	0.3 (5)
C20A—C21A—C22A—C23A	0.0 (6)	C20B—C21B—C22B—C23B	0.8 (5)
C21A—C22A—C23A—C8A	−1.5 (6)	C21B—C22B—C23B—C8B	−1.7 (5)
C7A—C8A—C23A—C22A	2.1 (5)	C7B—C8B—C23B—C22B	1.6 (5)
C9A—C8A—C23A—C22A	−176.2 (3)	C9B—C8B—C23B—C22B	−176.9 (3)

### Hydrogen-bond geometry (Å, º)

Cg1 is the centroid of the C11A–C16A ring.

**Table:**

*D*—H···*A*	*D*—H	H···*A*	*D*···*A*	*D*—H···*A*
O1*A*—H1*A*···O1*B*	0.84 (6)	1.96 (6)	2.777 (4)	162 (6)
O1*B*—H1*B*···*Cg*1^i^	0.83 (6)	3.18 (6)	3.959 (4)	158 (6)

Symmetry code: (i) *x*−1/2, −*y*+1, *z*.
